# Retail-based healthy food point-of-decision prompts (PDPs) increase healthy food choices in a rural, low-income, minority community

**DOI:** 10.1371/journal.pone.0207792

**Published:** 2018-12-12

**Authors:** Christopher R. Gustafson, Rachel Kent, Michael R. Prate

**Affiliations:** 1 Department of Agricultural Economics, University of Nebraska-Lincoln, Lincoln, Nebraska, United States of America; 2 Rosebud Food Sovereignty Initiative, Mission, South Dakota, United States of America; UT School of Public Health, UNITED STATES

## Abstract

This study examines the potential for point-of-decision prompts (PDPs) to promote healthier food choices among shoppers in a rural, low-income, minority community. We hypothesized that a narrowly defined PDP (focused on fresh produce) would be easier for shoppers to remember than a broadly defined PDP (focused on any healthy items), resulting in a higher proportion of healthy items purchased. PDPs were placed at the entrance to a supermarket in Mission, South Dakota, United States of America, on the Rosebud Sioux Reservation for alternating time periods, July 9–10, 2017. Sales records from 653 transactions were retrieved from the supermarket, comprising periods in which PDPs were in place and control periods. We examined the proportion of selected items and proportion of total expenditures that were a) any healthy foods and b) fresh fruits and vegetables. Data were analyzed in 2018. The narrowly defined prompt consistently resulted in a higher proportion of items and expenditures on healthy foods than either the broad prompt or the control condition. Shoppers in the narrow prompt condition purchased and spent significantly more on any healthy foods and fresh produce than shoppers in the control condition. While shoppers in the narrow prompt condition purchased more healthy foods and fresh produce than shoppers in the broad prompt condition, the differences were not statistically significant. Shoppers exposed to the narrow PDP consistently purchased more healthy foods than shoppers in a control group, while shoppers in the broad PDP did not, highlighting the importance of considering cognitive processes when designing health promotion messages.

## Introduction

Obesity, which has increased steadily in the US in recent decades [1], is linked to diverse negative consequences, which include poorer health; increased risks of non-communicable diseases, such as type-2 diabetes, various cancers, and heart disease; and decreased life expectancy [2]. Obesity results in higher healthcare costs and other adverse economic effects, such as higher rates of absenteeism [3], and can reduce individuals’ quality of life through various pathways, including experiences of social stigma, lower self-esteem, depression, and decreased physical function [4].

Obesity is not distributed evenly throughout the US population. Certain populations, including members of some ethnic groups as well as rural and poorer individuals, experience higher rates of obesity on average [5,6]. African American and Hispanic populations have markedly higher rates of obesity than white individuals of the same age groups [5,7]. American Indians are one of the highest risk groups for obesity [8]. American Indians frequently live in rural areas, face high levels of poverty and have limited access to markets with adequate offerings of healthy foods, conditions frequently linked to higher rates of obesity [6].

Sustained improvement of dietary quality is a priority for reducing obesity and diet-related health problems. However, access to healthy foods is not necessarily sufficient to improve dietary quality. Even when individuals gain access to healthy foods, food-purchasing patterns may not change [9]. A burgeoning line of research has focused on ways to encourage healthier behaviors at the point of decision, both in food choice and physical activity [10–12]. Point-of-decision prompts (PDPs) aim to disrupt habitual, unhealthy choices—by informing or persuading people—at the moment they are making a choice. PDPs may be particularly impactful in food choice, which relies more on autonomic, or unconscious, systems than many other decisions [13].

Efforts to promote healthier food choices at the point of decision have relied primarily on providing information, though there is a small literature examining financial incentives [14,15]. Nutrition information on packaged food products has been available for approximately 25 years (Federal Register, 58FR2079; Jan. 6, 1993), and the Affordable Care Act (2010) included a requirement that restaurants and other food retail outlets with at least 20 locations must provide nutritional information for prepared foods (Federal Register, 82 FR 20825; May 4, 2017), which was implemented May 7, 2018.

Research on the effect of information-based PDPs on food choice shows mixed results. The effect of nutrition facts panels on the quality of food choice in retail situations appears to be fairly minor, though improvements in certain dietary nutrients were observed [16]. Similarly, there is little evidence that providing nutrition information has a major impact on food choice in restaurants, particularly among low-income households [17]. However, simplified nutrition information included on front-of-pack or shelf-based labels shows more promise [18]. Research suggests simplified packages may facilitate the processing of information by consumers who face cognitive or time constraints in the store [19].

Cognitive load, a state in which an individual’s cognitive system is challenged by complex decisions, attempting to retain information, or other stressors [20], can influence decision-making. For example, cognitive load has been found to have a negative effect on healthy choices. Individuals choose more unhealthy foods under an induced high cognitive load [21], and are more impatient [22], which is related to less healthy decisions. Cognitive load and unhealthy decisions may be exacerbated by poverty because the poor repeatedly face stressful financial constraints [23]. While there are no studies that explicitly examine the interaction of cognitive load with the design of healthy food promotional materials, two recent studies on incentives indicate that the effectiveness of the incentive for low-income shoppers may be influenced by how the incentives are designed [14,15], which may be due to cognitive load.

A few studies suggest that PDPs that explicitly prompt individuals to consider health when making food decisions—in contrast to PDPs that simply present objective information—lead to healthier choices [14,24,25]. Research combining neural and behavioral evidence on food choice may illustrate why. When individuals are prompted to consider health when making a food choice, they not only choose healthier items but also exhibit different patterns of neural activation [24]. Additionally, evidence suggests that health information is naturally assimilated and processed more slowly than taste information [26], but increasing the salience of health information accelerates the integration of health information [27], which increases individuals’ responsiveness to health attributes [28].

In this study, we report the results of a grocery store-based field experiment that examines the efficacy of PDPs that explicitly encourage shoppers to consider health while making food choices in a predominantly low-income, minority population in the rural Midwest. Because low-income individuals are more likely to experience cognitive load when shopping than higher-income individuals [23], we test two prompt conditions that may interact differently with naturally occurring cognitive load to influence the effectiveness of the prompt. The two conditions that we test are a narrowly defined prompt (N-PDP) and a broadly defined prompt (B-PDP). The N-PDP encouraged shoppers to set a goal of purchasing at least five fresh fruits and vegetables (F&V), while the B-PDP encouraged shoppers to aim to purchase at least five healthy food items.

Since the items highlighted in the narrow prompt are a subset of the items in the broad prompt, it will be easier for shoppers to find preferred healthy food items within the broader set. However, if individuals experience cognitive load, it may be easier to keep the narrow prompt in mind while shopping. We hypothesize that exposure to any PDP (narrow and broad PDPs pooled together) will increase purchases of healthy foods relative to a control condition. Additionally, we hypothesize that shoppers in the N-PDP condition will increase their purchases of healthy foods more than shoppers in the B-PDP condition, and that purchases of healthy foods in each intervention condition will be higher than items purchased by shoppers in a control condition.

## Methods

The University of Nebraska-Lincoln’s Institutional Review Board approved this research (#20170216932EX) and Rosebud tribal authorities approved the research. Neither written nor oral consent was obtained because researchers did not interact with research subjects or collect personally identifiable information. The University of Nebraska-Lincoln's IRB waived the informed consent requirement.

The research was conducted in a supermarket on the Rosebud Indian Reservation in Mission, Todd County, South Dakota. The UNL IRB waived the informed consent requirement. The Rosebud Indian Reservation is the home of the Rosebud Sioux Tribe—over 90% of Reservation residents are fully or partially Native American [29]. Residents of the Rosebud Indian Reservation have many of the characteristics that are associated with poorer health outcomes. Poverty is widespread. The per-capita income in 2016 was under $12,000, and nearly 50% of residents lived in poverty [29]. Additionally, educational attainment is markedly lower than the US average, with less than 15% of adults having completed college [29]. Access to food retail outlets is also limited. There are only two other grocery locations on the reservation, though both are open daily. One is approximately two miles from the study supermarket in Mission, while the third is ten miles away in Rosebud. All of these factors are associated with lower quality diets and poorer health.

The supermarket experiment was conducted on consecutive days, a weekend day and a weekday, July 9–10, 2017. The research was limited to two days for two reasons. Most importantly, store management only agreed to provide data on purchases for two days due to the logistically and time intensive nature of generating purchase data. The data were only accessible by printing out itemized receipts and obtaining data for two days resulted in approximately 1,000 printed pages. Second, researchers only had access to one supermarket. To get a clean measure of the effect of the intervention, we needed to avoid exposing shoppers to multiple conditions on repeat visits, which could lead to a loss of control over consumer behavior in the experiment. Each condition was implemented for two hours each day and rotated so that one day the B-PDP condition occurred first, followed by a control condition, and finally the N-PDP condition, while the other day the conditions were reversed.

**Fig 1:**
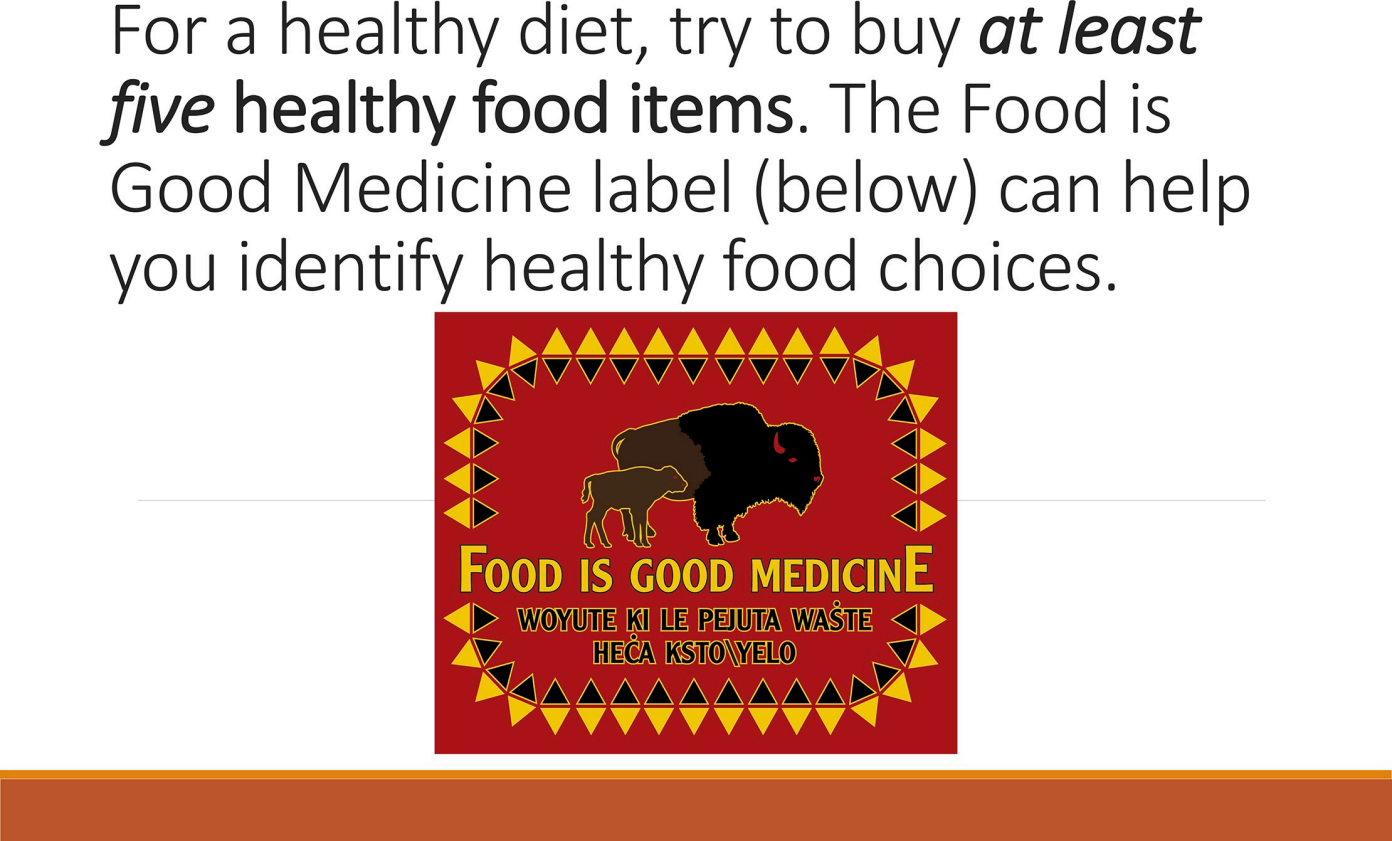
Poster with point of decision prompt for the broad prompt condition.

Narrow and broad prompt messages were printed on posters, which were two feet high by three feet wide. The posters were displayed on an easel just inside the sole entry point into the supermarket so that all shoppers entering the store passed by the posters. Easels were set to be in the line of sight of shopper with the bottom edge of a poster 3.5 feet above the ground. We used gain-framed messages, rather than loss-framed messages, in designing the PDPs. Gain-framed messages highlight positive aspects of the change in behavior that is being encouraged, while loss-framed messages focus on the costs of continuing the behavior. In the context of food choice, gain-framed messages emphasize healthier dietary choices and better health. Loss-framed messages, on the other hand, would stress the risks that poor diets would pose to the individual’s health. We selected gain-framed messages for the PDPs because they have been found to be more effective in promoting healthy behaviors than loss-framed messages [30]. The N-PDP condition message read “*For a healthy diet*, *try to buy at least five fruits and vegetables*. *Food is Good Medicine*.” The B-PDP message read “*For a healthy diet*, *try to buy at least five healthy food items*. *The Food Is Good Medicine label can help you identify healthy food choices*” (Fig 1). The number of items to include in the prompt was selected based on consultation with the store manager and represented twice the average number of healthy items purchased per transaction prior to the experiment. Both messages refer to a healthy food label featuring the phrase “Food Is Good Medicine” (FIGM). The FIGM label was developed within the community to identify healthier food items and had been implemented in the supermarket nine months before the current study took place. A university-employed registered dietitian evaluated the supermarket inventory to establish which items to label as healthy. All items promoted in the prompts carried the FIGM label so that shoppers would be able to easily identify healthy choices in both N-PDP and B-PDP conditions.

Data on 653 transactions, with information on items purchased, price of each item, total number of items purchased, and total amount spent on items purchased, were analyzed in 2018. Exclusion criteria for transactions were: 1) if the shopper did not purchase any food items, or 2) if the shopper purchased only single-serving, ready-to-eat foods and/or beverages from the supermarket deli. Non-food items were subtracted from the total item count for receipts that included food and non-food items. Researchers coded each purchased food item as healthy or unhealthy based on the FIGM system and recorded the total number of all food items, all healthy items, and all F&V as well as expenditures on all food items, on all healthy items, and on F&V per transaction.

Researchers combined the data on the number and amount of money spent on all healthy foods and on F&V with data on total food items purchased and total amount spent on food to create variables that capture all healthy items or all F&V as a proportion of all items purchased and of total food expenditures. Proportions control for variation in the number of items purchased and amount spent from one transaction to another. We used these variables as our key outcomes to evaluate the effect of the pooled PDPs and the separate N-PDP and B-PDP conditions. Because the data were generated by the supermarket’s sales management software, we do not have access to typical demographic control variables, such as gender, education, age, or income. However, we also avoid concerns about researcher demand effects since researchers did not interact with shoppers during the research [31]. As we compared multiple conditions, we used Bonferroni correction for multiple comparisons to adjust tests of statistical significance. Data were analyzed using R Statistical Software (R Core Team, 2018). We considered p-values ≤ 0.05 to be statistically significant.

## Results

First, we compare the pooled PDP purchases to purchases in the control condition (Table 1). The four outcomes of interest are the proportion of all food items purchased that were healthy, the proportion of all food items purchased that were fruits and vegetables, the proportion of all expenditures spent on any healthy foods, and the proportion of all expenditures spent on fruits and vegetables. We also tested whether the day of the week affected the outcomes of interest. We found no statistically significant relationship between day of the week and healthy food choices. Additionally, estimates of the effect of the PDP on food choices did not change with the inclusion of a day of the week variable, so we omit the day of the week variable from the results we report.

**Table 1:** Purchases of healthy items in pooled prompt condition versus control condition.

	Condition		% Difference
Measure	PDP Mean (SD)	Control Mean (SD)	PDP–Control
**Healthy Products Purchased**	0.156 (0.217)	0.114 (0.157)	36.8**
**Fruit & Vegetables Purchased**	0.095 (0.184)	0.068 (0.122)	39.7*
**Expenditures on Healthy Products**	0.135 (0.208)	0.099 (0.145)	36.4*
**Expenditures on Fruit & Vegetables**	0.087 (0.178)	0.063 (0.115)	38.1*
**N**	327	326	

Notes: Means represent the fraction of each measure (items and expenditures on any healthy item and fruits and vegetables). Significance is calculated using pairwise t-tests with Bonferroni adjustment for multiple comparisons.

(* p<0.05; ** p<0.01).

The proportion of healthy items purchased by shoppers in the control condition (no PDP displayed) was 0.114. For shoppers in a PDP condition, the proportion was 0.156. This represents a 37% increase in the proportion of healthy food items purchased and is statistically significant (p<0.01). For fresh fruits and vegetables, the proportion purchased increased from 0.068 in the control condition to 0.095 in the PDP condition (p<0.05). The proportion of food expenditures demonstrates very similar patterns. Expenditures on all healthy items increased from 0.099 in the control condition to 0.135 in the PDP condition (p<0.05), while expenditures on fruits and vegetables increases from 0.063 in the control condition to 0.087 (p<0.05). In each case, the proportion of healthy purchases or expenditures is between 36 and 40 percent higher in the PDP condition.

We break the pooled PDP data into the narrow (N-PDP) and broad (B-PDP) message conditions to evaluate whether one condition drove the increase in the proportion of healthy purchases observed in the first analysis. Again, we compare purchases in the two PDP conditions to the control condition (Table 2). The proportion of healthy items purchased by shoppers in the control condition was, again, 0.114. After disaggregating the PDP conditions, we see that in the B-PDP condition, the proportion of healthy items purchased items was 0.138, while in the N-PDP condition, it was 0.170. The difference between N-PDP and the control condition was statistically significant (p<0.01).

**Table 2:** Purchases of healthy foods in N-PDP, B-PDP, and control conditions.

	Condition			% Difference		
Measure	N-PDP Mean (SD)	B-PDP Mean (SD)	Control Mean (SD)	N-PDP–B-PDP	N-PDP–Control	B-PDP–Control
**Healthy Products Purchased**	0.170 (0.220)	0.136 (0.211)	0.114 (0.157)	25.0	49.1**	19.3
**Fruit & Vegetables Purchased**	0.109 (0.193)	0.075 (0.168)	0.068 (0.122)	45.3	60.3*	10.3
**Expenditures on Healthy Products**	0.151 (0.219)	0.112 (0.189)	0.099 (0.145)	34.8	52.5*	13.1
**Expenditures on Fruit & Vegetables**	0.102 (0.196)	0.067 (0.150)	0.063 (0.115)	52.2	61.9*	6.3
**N**	189	138	326			

Notes: Means represent the fraction of each measure (items and expenditures on any healthy item and fruits and vegetables). Significance is calculated using pairwise t-tests with Bonferroni adjustment for multiple comparisons.

(* p<0.05; ** p<0.01).

Next, we examine the proportion of F&V purchased by condition. In the control condition, 0.068 of shoppers’ items were F&V. In the B-PDP condition, the proportion was 0.075, while in the N-PDP condition, the proportion was 0.109. The proportion of F&V purchased in the N-PDP condition was significantly higher than in the control condition (p < 0.03).

Expenditures on healthy items follow a pattern similar to the number of items purchased. In the control condition, the proportion of expenditures on healthy food items was 0.099, while the proportion of expenditures on healthy foods was 0.112 in the B-PDP condition. Shoppers in the N-PDP condition spent a significantly higher proportion, 0.151, of total expenditures on healthy items than shoppers in than the control condition (p = 0.01).

The proportion of expenditures on F&V is slightly lower than the proportion of F&V purchased but shows the same relationship across conditions. The lowest proportion of total expenditures on F&V occurred in the control condition (0.063), followed by the B-PDP condition (0.067). In the N-PDP condition, the proportion spent on F&V was highest: 0.102. Expenditures on F&V in the N-PDP condition were significantly higher than the proportion of F&V expenditures in the control condition (p < 0.05).

We use one additional measure to assess whether shoppers’ responses to the PDPs are consistent with the PDP’s message. If shoppers respond to the prompt, shoppers in the N-PDP condition should increase purchases of F&V relatively more than shoppers in B-PDP. For this measure, we calculate the change in each of the four variables we examined above between the control and both PDP conditions. We calculated this measure for all four variables and for both PDP conditions. We then look at the percentage of the difference in healthy food purchases/expenditures attributable to fruits and vegetables. For instance, we take the proportion of F&V purchased in N-PDP minus the proportion of F&V purchased in the control condition, and the proportion of all healthy foods purchased in N-PDP minus the proportion of all healthy foods purchased in the control condition. We then divide the difference in F&V by the difference in all healthy foods. The results suggest that shoppers did respond differently to the two prompts (Table 3). In the B-PDP condition, 31% of the difference in healthy food purchases relative to the control condition is attributable to F&V, while the remaining 69% comes from other healthy foods. In the N-PDP condition, 75% of the difference in healthy food purchases comes from F&V, while the remaining one-quarter is attributable to other healthy foods.

**Table 3:** Increase in proportion of all healthy foods and fruits and vegetables purchased in N-PDP and B-PDP relative to control.

	N-PDP–Control	B-PDP–Control
**Number of items purchased**		
**Fruits and Vegetables (F&V)**	0.041	0.007
**All Healthy Foods (AHF)**	0.056	0.022
**F&V/AHF (%)**	73%	31%
**Expenditures**		
**Fruits and Vegetables (F&V)**	0.039	0.004
**All Healthy Foods (AHF)**	0.052	0.013
**F&V/AHF (%)**	75%	31%

## Discussion

Concerns about the relationship between diet, obesity and health outcomes have arisen in recent decades, especially for low-income populations. Most efforts to change behavior at the point of decision have provided information about the nutritional content of packaged and prepared foods. The majority of research on information-based PDPs find no effect [16,17]. The limited effectiveness of information-based PDPs may result from the shopper’s cost of accessing, interpreting, and using information [18,19]. However, prompting shoppers to explicitly consider health can increase their purchases of healthy foods [24,25]. This approach appears to change how health information is integrated and weighted when making choices [27,28].

In our grocery store-based field study, we examined the effect of two PDP prompts encouraging shoppers to set a healthy food purchase goal. The shoppers were from a low-income community with high rates of obesity and diet-related health problems, and the prompts were designed to test whether interaction between naturally occurring cognitive load and the prompt message influences PDP effectiveness. A narrow prompt encouraged shoppers to set a goal of purchasing five F&V (N-PDP), while the broad prompt condition encouraged shoppers to set a goal of purchasing any five healthy items (B-PDP). We consistently find that shoppers purchase the greatest amount and spend the most on healthy food items in the N-PDP condition. While the number of items purchased and amount spent in the B-PDP condition for healthy foods is higher than the control condition, the differences are not statistically significant. Shoppers in the N-PDP condition purchased a higher proportion of healthy foods overall (49% higher) and F&V (60.3% higher) than the control condition. The N-PDP condition yielded more purchases than the B-PDP condition of all healthy foods (25% higher) and F&V (45% higher). Expenditures show a similar pattern. Shoppers in the N-PDP condition spent more than shoppers in the control condition on all healthy items (52.5% higher) and on F&V (61.9% higher). Shoppers also spent a higher percentage in the N-PDP condition than in the B-PDP condition (34.8% higher for all healthy foods and 52.2% higher for F&V).

The observed patterns support our hypotheses about the interaction between prompt design and cognitive load. Shoppers in the N-PDP consistently purchased more F&V than shoppers in the B-PDP or control condition, leading to higher overall purchases of healthy items. Shoppers in the B-PDP did not increase purchases of other healthy items enough to keep pace with the increases in healthy purchases of shoppers in N-PDP.

Our findings yield multiple important points. First, a simple, inexpensive, poster-based prompt can lead to an immediate, significant increase healthy food purchases in a low-income community at high risk of obesity and diet-related diseases. This is important because research findings frequently suggest that it is difficult to change the shopping habits of low-income consumers [9,15], though the data do not allow us to say whether these changes are sustained over time. Second, the PDP messages were overlaid on pre-existing informational PDPs, illustrating the importance of designing PDPs that go beyond simply providing information to targeting motivation or goals. Packaged products in the supermarket already contain nutrition facts panels, which have been defined as an informational PDP [11]. There is also a healthy food labeling system in the supermarket that identifies healthier products. Simple healthy food labels have been shown to be more effective than detailed nutrition information when shoppers face constraints [19], though it does not explicitly encourage shoppers to consider health. While this research does not explicitly test our messages against the existing information-based PDPs, which are present in all three conditions, the results suggest that PDPs that encourage consideration of health promote healthy choices beyond prompt strategies that rely on providing information to consumers [24]. Thus, optimal design of PDPs may require multiple layers of informational and motivational prompts.

Third, our results suggest that the design of prompts and other point-of-decision materials needs to consider the possibility of a target audience experiencing cognitive load. We find that shoppers purchase a significantly higher percentage of healthy foods and fresh fruits and vegetables when exposed to a PDP message focused on a limited set of products (N-PDP) compared to a control group, while shoppers exposed to a PDP message encouraging the purchase of a broader range of products (B-PDP) did not significantly increase their healthy food purchases. This is despite the fact that shoppers have access to more products available for purchase in the B-PDP condition than in the N-PDP condition—since items identified in N-PDP are a subset of items in B-PDP. This finding may result from the narrow prompt being easier to keep in mind than the broad prompt if shoppers experience cognitive load.

There are limitations to this study, which require further research. The most significant limitation is the duration of the study. Our results demonstrate short-term impacts of the PDPs in a field setting, but future research should investigate long-term effects of these PDPs, which would require implementation across multiple stores. Research suggests that information-based PDPs may be overlooked once individuals become habituated to the materials [17], though other studies do not find a reduction in effect over time [32]. Further research examining whether the effect of PDPs that makes health-related goals salient is attenuated over time is necessary to know whether the changes that we observe in these data would continue in the long-run. Social desirability bias, which occurs when participants in a study behave in a way that they think will be perceived positively more than they would if they did not feel that their behavior were under scrutiny, may also influence behavior. While the study design minimizes the influence of social desirability bias by eliminating interaction between researchers and study subjects—which is the classical source of social desirability bias, it is possible that shoppers changed their behaviors knowing that they would interact with supermarket employees when purchasing their items. Future research could examine shopper-salesperson interactions as a source of social desirability bias by studying differences in choices of shoppers who go through conventional—i.e. employee staffed—versus self-checkout lanes.

Despite study limitations, this research identifies a simple, low-cost PDP that significantly increased the relative purchase of and amount spent on healthy foods and on fresh fruits and vegetables in a low-income community at high risk of obesity and diet-related diseases. Though there is little preliminary research on PDPs that encourage individuals to consider health when making shopping decisions, these results identify the approach as a potentially effective, inexpensive means by which to increase the healthfulness of retail food purchases.

Our study provides evidence of the feasibility of using a low-cost point-of-decision prompt (PDP) to increase healthy food purchases in a low-income, minority community. Our research also emphasizes the importance of considering the impact of cognitive stressors—such as those introduced while individuals are actively thinking about what they need to buy and how much they can spend—on the effectiveness of the PDP. These results suggest that PDPs may need to be carefully designed to help shoppers retain and respond to the message.
